# The renin-angiotensin-aldosterone system (RAAS) signaling pathways and cancer: foes versus allies

**DOI:** 10.1186/s12935-023-03080-9

**Published:** 2023-10-27

**Authors:** Bahareh Hassani, Zeinab Attar, Negar Firouzabadi

**Affiliations:** 1 Medicinal and Natural Products Chemistry Research Center, Shiraz University of Medical Sciences, Shiraz, Iran; 2 Recombinant Proteins Department, Breast Cancer Research Center, Motamed Cancer Institute, ACECR, Tehran, Iran; 3 Department of Pharmacology & Toxicology, School of Pharmacy, Shiraz University of Medical Sciences, Shiraz, Iran

**Keywords:** Renin-angiotensin-aldosterone system, Cancer biology, ACE inhibitors, ARBs, sex hormone-dependent cancer, GI cancer

## Abstract

The renin-angiotensin-aldosterone system (RAAS), is an old system with new fundamental roles in cancer biology which influences cell growth, migration, death, and metastasis. RAAS signaling enhances cell proliferation in malignancy directly and indirectly by affecting tumor cells and modulating angiogenesis. Cancer development may be influenced by the balance between the ACE/Ang II/AT1R and the ACE2/Ang 1–7/Mas receptor pathways. The interactions between Ang II/AT1R and Ang I/AT2R as well as Ang1-7/Mas and alamandine/MrgD receptors in the RAAS pathway can significantly impact the development of cancer. Ang I/AT2R, Ang1-7/Mas, and alamandine/MrgD interactions can have anticancer effects while Ang II/AT1R interactions can be involved in the development of cancer. Evidence suggests that inhibitors of the RAAS, which are conventionally used to treat cardiovascular diseases, may be beneficial in cancer therapies.

Herein, we aim to provide a thorough description of the elements of RAAS and their molecular play in cancer. Alongside this, the role of RAAS components in sex-dependent cancers as well as GI cancers will be discussed with the hope of enlightening new venues for adjuvant cancer treatment.

**Graphical abstract:**

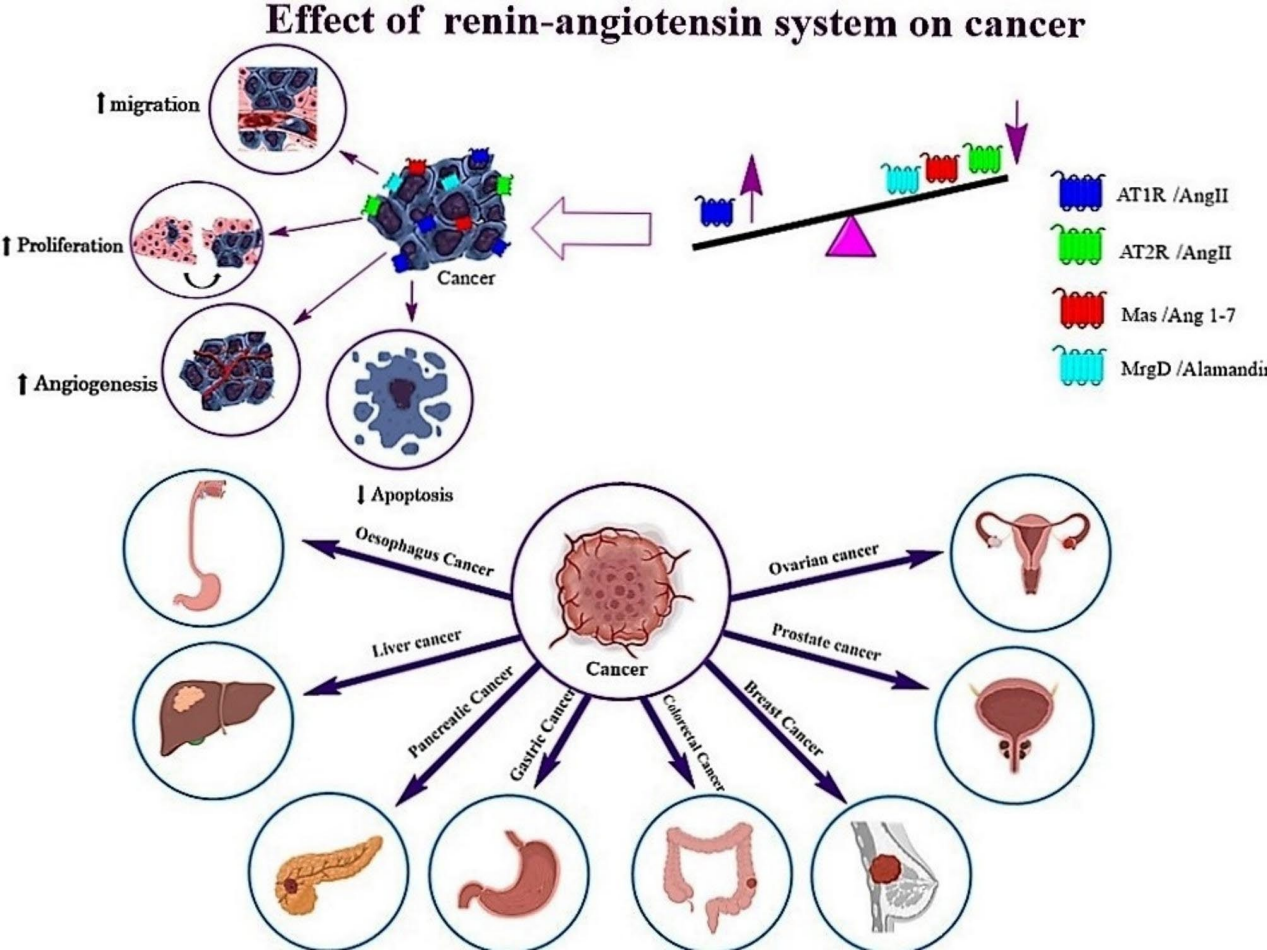

## Introduction

Cancer is the greatest clinical, social, and economic burden measured in cause-specific Disability-Adjusted Life Years (DALYs). The accelerating prevalence of cancer and the rising expense of cancer therapy highlight the urgent need for more efficient and cost-effective therapies [1]. Nearly every organ in the human body expresses functional RAAS at various levels [2]. The relationship between RAAS signaling and cancer has recently been highlighted. RAAS signaling is a key player in maintaining many physiological conditions [3]. Dysregulation of RAAS signaling frequently happens in cancer. Indeed, changes in the function and activity of RAAS elements play various roles in carcinogenesis [4]. Numerous tumor types, including sex hormone-dependent cancers such as breast, prostate, cervical and ovarian cancers as well as GI cancers like pancreatic, stomach, liver and colon cancer along the cancers of the brain, lung, skin, and hematopoietic cells have been shown to exhibit increased RAAS activity [5, 6]. The involvement of RAAS in cell proliferation, inflammation, metastasis, and angiogenesis has recently received great attention. Hence, applying therapeutic options which target the RAAS pathways might inhibit tumor growth and metastasis, and increase overall survival.

Receptors and effectors in the RAAS pathway can have opposite effects in preventing or developing cancer. Therefore, a better understanding of the RAAS signaling pathway and the effective factors and drugs affecting each of the receptors can lead to new therapeutic options. RAAS proteins are small peptides with opposing effects on cancerous cells. Angiotensin II (Ang II), the major peptide of the classical axis is a product of the angiotensin-converting enzyme (ACE). It has various effects on affecting AT1R and AT2R. Ang II enhances cell proliferation and angiogenesis through the signaling of AT1 receptor, whereas Ang II induces anti-proliferative effects through AT2 receptor [7]. Angiogenesis, fibrosis, increased tumor invasion, and metastasis can all be attributed to the binding of Ang II to AT1R. Contrarily, when Ang II binds to AT2R, it causes opposite effects that may prevent cancer [8]. The ACE2/Ang1-7/Mas axis is described as the counter-regulatory axis of the classical ACE/Ang II/AT1R axis. Ang 1–7, the peptide produced by ACE2, generally inhibits cell proliferation via Mas receptors [9]. Ang 1–7/Mas axis along with the Ang II/AT2R axis are antagonists of the classic axis of ACE/Ang II/AT1R, particularly under pathological conditions [10]. Ang 1–7 has anti-inflammatory, anti-fibrotic, anti-proliferative, and anti-migratory effects when it binds to the Mas receptor [8]. Therefore, RAAS pathway can have dualistic effects.

Here we emphasized on the dualistic role of RAAS in cancer. We discussed the elements of the RAAS pathways and their role in sex-hormone-dependent cancers (breast, ovary, and prostate) as well as common GI cancers. Moreover, drugs affecting RAAS along with their potential role in the above-mentioned malignancies are discussed.

## The RAAS pathway

Along with the prominent function of the renin-angiotensin-aldosterone system as the most important blood pressure regulator, RAAS could be related to different pathological conditions such as cancer and oncogenesis [11]. RAAS is mainly regulated by the heart and kidney, and its main function is to maintain body homeostasis and tissue perfusion. This cooperation is done in such a way that allows the kidney to maintain blood volume and the body’s water content whereas the cardiovascular system supplies blood circulation [12]. Angiotensin II (Ang II), a potent vasoconstrictor, is produced by the RAAS pathway with the aid of a sequence of enzymatic processes. Researchers have discovered the existence of RAAS in various organs and tissues such as the gastrointestinal and nervous system, liver, muscle, bone, pancreas, and adipose tissue [13].

The knowledge of the traditional RAAS has progressed through identification of new actors, Angiotensin-converting enzyme 2 (ACE2), prorenin receptor (PRR), and the Mas receptor [14]. New perspectives on the physiology and pathology of the RAAS have been achieved by proposing the RAAS pathway as a two-axis system consisting of the Angiotensin-converting enzyme (ACE)/Ang II/Ang II type 1 receptor (AT1R) and ACE2/Angiotensin 1–7 (Ang 1–7)/Mas receptor [15, 16], along discovering PRR and its physiologic function [17, 18], as well as discovering angiotensin 1–9 (Ang 1–9) as an active protein in the RAAS [19, 20]. These new perspectives on the RAAS enable researchers to target RAAS to treat various diseases [14].

### Angiotensinogen and angiotensin derivatives

Angiotensinogen can be considered the precursor of other Angs. In fact, angiotensinogen is a glycoprotein which is the starting point of the RAAS pathway. There are 485 amino acids in human angiotensinogen which include a signal peptide of 33 amino acids. Angiotensinogen belongs to the non-inhibitory serpin superfamily which are serine protease inhibitors [21]. Production and secretion of Ang occurs in the liver, but it should be noted that although the liver is the main producing organ, angiotensinogen is additionally produced in the brain, lungs, kidneys, vasculature, and heart [12]. Renin can cleave the N-terminal amino acids of angiotensinogen to generate the inactive decapeptide Ang I. Ang I can be considered a source of active Ang peptides. The rate-limiting step for the release of Ang I is the cleavage of angiotensinogen by renin [21]. Renin cleavage of angiotensinogen is different in various species [22, 23].

**Fig. 1:**
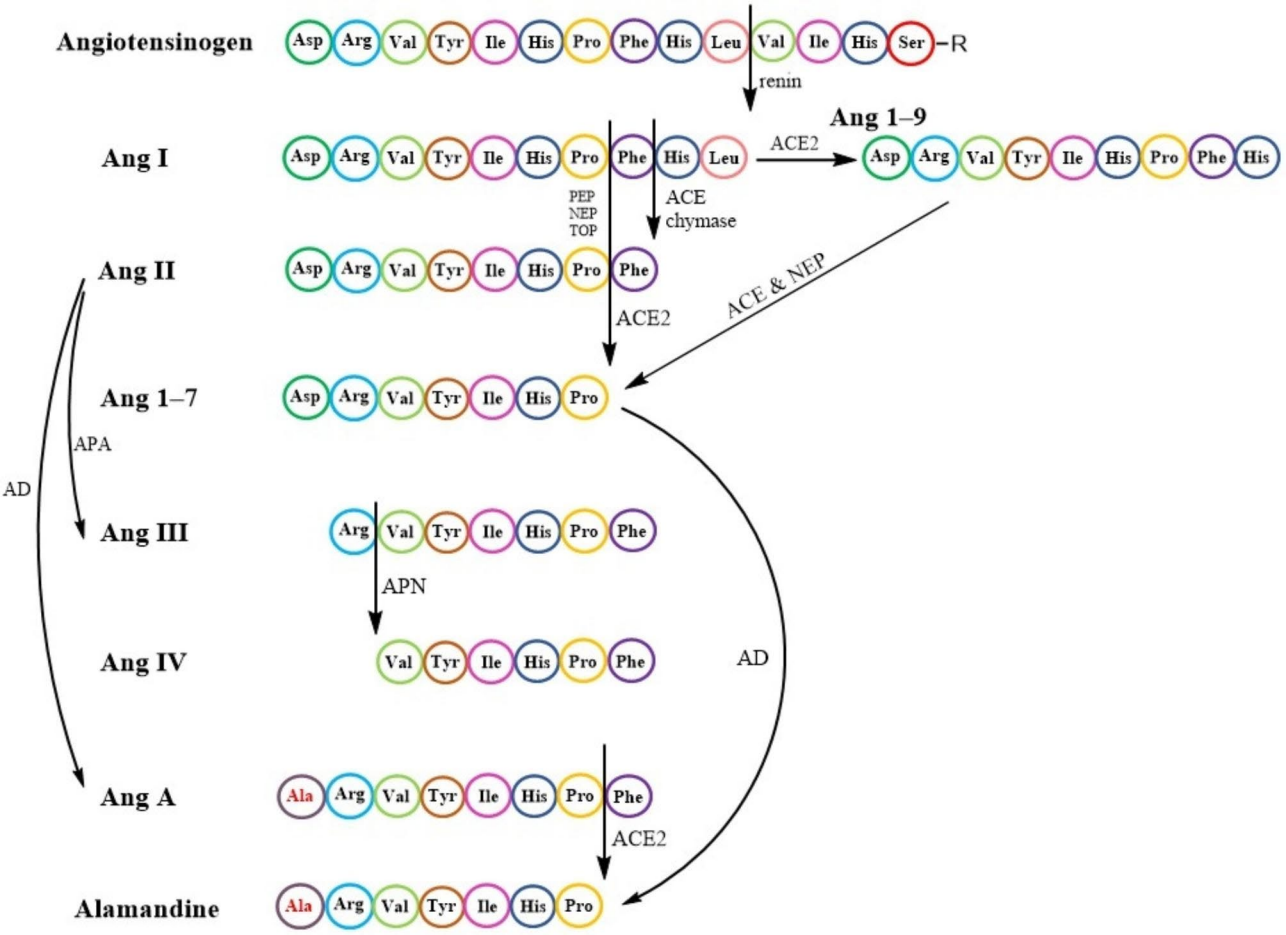
RAAS peptides, receptors and enzymes Angiotensinogen is the precursor of all angiotensin derivatives. Angiotensinogen can be cleaved by renin to produce Ang (I) Ang I cleaves into Ang II by ACE or chymase. Some peptidases, like TOP, NEP, and PEP, can convert Ang I into Ang 1–7. ACE2 can hydrolyze Ang II to create Ang 1–7 as well. In addition, Ang I can be cleaved by ACE2 to create Ang 1–9, which can be cleaved by ACE or NEP to generate Ang 1–7. Ang III (Ang 2–8) can be driven from Ang II by APA. As Ang III is further cleaved, it produces Ang IV (Ang 3–8). Through the decarboxylation of Asp1, Ang A is produced from Ang (II) Ang A could be converted to alamandine by ACE2. Alamandine can also be produced by decarboxylation of the aspartate residue in Ang 1–7

An overview of the angiotensin derivatives is displayed in Fig. 1. Ang I cleaves into an active octapeptide called Ang II. This cleavage is carried out by ACE or chymase. Ang 1–7 can be derived from Ang I or Ang II through endo-peptidases or carboxy-peptidases respectively. Ang I can be converted into Ang 1–7 by some peptidases for instance TOP (thimet oligopeptidase), NEP (neutral endopeptidase), and PEP (prolyl-endopeptidase). The Pro^7^-Phe^8^ linkage in Ang I can break and convert Ang I to Ang 1–7. Ang II is also able to be hydrolyzed by ACE2 to produce Ang 1–7 [24, 14]. In addition, Ang I can be cleaved by ACE2 to create Ang 1–9, which can be cleaved by ACE or NEP to generate Ang 1–7 [25, 14]. In other words, ACE2 can cut phenylalanine amino acid from Ang II and convert it to Ang 1–7. Furthermore, Ang I can be converted to Ang 1–9 by ACE2 by removing the amino acid leucine. Therefore, ACE2 appears to be crucial for the formation of Ang 1–7 and Ang 1–9 [26, 27]. Ang III (Ang 2–8) is another Ang derivative that is derived from Ang II by aminopeptidase A (APA). It has the ability to bind to AT1R and induce the same pressor response as Ang II, but with a more significant effect on the brain [28]. As Ang III is further cleaved, it produces Ang IV (Ang 3–8), which can interact with AT4R and promote vasodilation in the cerebral and renal vascular beds and facilitate sodium excretion and renal blood flow [29]. Another derivative of Ang is Ang A (Ang A) which is considered a physiologically active metabolite of Ang II. Ang A is an octapeptide whose amino acid sequence only differs by one amino acid from Ang II. Thus, in Ang A, there is an Ala^1^ amino acid instead of Asp^1^. Ang A probably generates from Ang II via decarboxylation of Asp^1^. Ang A can interact with both types of AT1R and AT2R receptors [30]. Alamandine could be produced from Ang A by ACE2. Additionally, decarboxylation of the aspartate residue in Ang 1–7 can also generate alamandine directly. In fact, Alamandine simply varies from Ang 1–7 in that its N-terminal residue is an alanine rather than an aspartate [31]. Alamandine can exert its effects by interacting with Mas-related G protein-coupled receptor D (MrgD). Therefore, the Ang A/alamandine/MrgD signaling pathway emerges [32]. Research indicates that the function of various organs and systems could be related to the balance between the activation of the ACE/Ang II/AT1R and the ACE2/Ang 1–7/Mas receptor pathway. Factors that cause imbalance towards the ACE/Ang II/AT1R pathway can result in cardiovascular diseases. It is worth mentioning that the function of this axis is beyond the cardio-renal and vascular due to its pleiotropic effects and can affect a wide range of diseases [33, 34, 35]. Inflammation, oxidative stress, fibrosis, and hypertrophy are associated with the activation of the ACE/Ang II/AT1R pathway [36].

### Angiotensin 1–7

An active heptapeptide known as Ang 1–7 is another Ang derivative [36]. Ang 1–7 can interact with the endogenous Mas receptor which can antagonize the actions of Ang II and can counter-regulate most of Ang II’s negative effects. Therefore, it has positive cardiovascular effects and is able to decrease blood pressure. In addition to its initially identified cardiovascular and renal functions, Ang 1–7 has different functions in various tissues and organs [37]. Studies have shown that the Mas antagonist, A779, can block most of Ang 1–7’s actions [38, 39, 40, 41]. Furthermore, the effects of Ang 1–7 are not found in Mas-deficient animals [42, 40, 43, 44]. MrgD can also mediate some of the actions of Ang 1–7 [42, 40, 43, 44]. Therefore, it was concluded that the effect of Ang 1–7 can be through the Mas receptor. The Ang 1–7/Mas receptor interaction can regulate several signaling pathways, including the ERK and phosphoinositide 3-kinase (PI3K)/protein kinase B (AKT) pathways, as well as affect downstream effectors like NO, cyclo-oxygenase-2 (COX-2) and forkhead box O1 (FOXO1) [14]. These mechanisms allow Ang 1–7 to improve pathological disorders such as inflammation and fibrosis in various organs like the liver, lungs, and kidneys. Due to its potential to prevent angiogenesis and cell proliferation, Ang 1–7 can be considered a potential anticancer therapy [14].

### Renin and prorenin

Prorenin is a precursor of renin. This transformation occurs in juxtaglomerular granular cells of the renal macula densa. Juxtaglomerular granular cells are specialized smooth muscle cells found primarily in the walls of the renal afferent arterioles that are involved in the generation, storage, and release of renin [45]. Various conditions trigger renin secretion from the kidneys into the bloodstream among which a reduction in blood pressure is considered the most crucial factor. Other stimulators of renin secretion include sympathetic stimulators and humoral factors such as Ang II, vasopressin, NO, histamine, prostaglandins, endothelins, and dopamine [45, 46]. In addition to the kidney, the uterus, placenta, testicles, adrenal gland, retina, and submandibular glands also produce renin [47].

### ACE & ACE2

ACE is a dipeptide carboxypeptidase and is responsible for converting Ang I to Ang II. On the other hand, ACE2 is a carboxypeptidase that is responsible for converting Ang II to Ang 1–7 [59, 25]. Although ACE2 and ACE have considerable similarities, ACE inhibitors do not block ACE2 activity [34]. ACE2 is a transmembrane glycoprotein that has a mono-carboxy-peptidase function which has a catalytically active ectodomain. Although ACE2 is expressed in most tissues of the body, the highest expression of ACE2 is in the lungs, kidneys, heart, and endothelium [60, 61]. It can be considered that the major function of ACE2 is the production of Ang 1–7 from Ang II, which can antagonize the action of Ang II [34]. Although the main substrate of ACE2 is Ang II, other peptides with lower affinity can be degraded by ACE2 [61, 34]. ACE2 can lessen the deleterious effects of RAAS in a variety of ways. Firstly, it is responsible for the cleavage of Ang I and Ang II, therefore it can reduce the available substrate of the ACE/Ang II/AT1R pathway, and in addition, it is responsible for the production of Ang 1–7 so it can increase the available substrate of the protective ACE2/Ang 1–7/Mas receptor pathway [62].

### AT1R & AT2R

AT1R and AT2R are G protein-coupled receptors and have pivotal roles in the RAAS pathway. Both receptors have a similar affinity for Ang II [63, 64]. While AT1R’s activities have long been known, AT2R was only discovered recently, and many of its activities are still not completely understood [65, 66]. AT2R is effective in the protective arm of RAAS and has the potential to be targeted for drug development. It has beneficial effects in improving cardiovascular, renal, lung, neural, and cutaneous diseases in addition to cancer [67]. The maximum relative affinities for AT1R and AT2R are possessed by Ang II and Ang III, respectively [68]. Most of the harmful and negative effects of Ang II are through its effect on AT1R [69]. A variety of protective actions are mediated by AT2R in the pathophysiological conditions, which are very similar to the effects of Ang 1–7 via Mas receptor, and include immune modulation, anti-inflammatory, antiapoptotic, antifibrotic, neuro-regeneration actions and inhibition of sympathetic outflow [70, 36]. Activation of AT2R may inhibit cell proliferation, promote vasodilation, and reduce oxidative stress and inflammation [71]. The proper balance between AT1R and AT2R function has a significant effect on the physiological activities of major organs [72, 69].

### Prorenin receptor (PRR)

PRR is a transmembrane protein with three domains: an extracellular domain with the N-terminal region, a transmembrane domain, and a cytoplasmic domain with the C-terminal region. [48]. It is commonly agreed that PRR is an essential receptor for regulating RAAS. The primary function of PRR is Ang II formation [49]. This receptor was known primarily for its ability to enhance RAAS by interacting with prorenin, and renin and also activating the intracellular mitogen-activated protein kinase (MAPK)/ extracellular signal-regulated kinase (ERK) pathway independently of the RAAS, thus having a crucial effect on renal and cardiovascular function [50]. PRRs are implicated in different physiologic and pathologic conditions and pathways, including Vacuolar ATPase (V-ATPase) activity [51], Wnt/β-catenin signaling [52], Par3 system, and tyrosine-phosphorylation-dependent signaling pathways [49]. Latest research has demonstrated that benign tumors and human cancers express PRR much more than normal tissues. It was found to be overexpressed in breast cancer [53], glioma [54], pancreatic ductal adenocarcinoma [55, 56], aldosterone-producing adenoma [57], and colorectal cancer [58].

### Mas receptors

Mas receptor is a transmembrane protein and a member of G protein-coupled receptors [73]. There is a subtype of Mas receptor called Mas-Related G-Protein Coupled Receptor D (MrgD), with an affinity for alamandine [74]. Alamandine does not attach to the Mas receptor, however, it binds to the MrgD receptor with the same features [75]. Activation of the MAS receptor in the RAAS reduces chronic hypertension, fibrosis, blood pressure, and sympathetic tone and increases vasodilation, nitric oxide production, parasympathetic tone, baroreflex, and natriuresis [40]. Positive effects related to the Mas receptor are produced by its interaction with Ang 1–7 [76].

## The RAAS pathway and cancer

Nearly every organ in the human body expresses a functional RAAS at various levels [2]. Numerous tumor types, including cancer of the breast, kidney, pancreatic, prostate, stomach, bladder, cervix, brain, lung, liver, colon, skin, and hematopoietic cells, have been shown to have increased RAAS activity [5, 6, 5]. The involvement of RAAS in cell proliferation, inflammation, metastasis, and angiogenesis has received more attention recently.

**Fig. 2:**
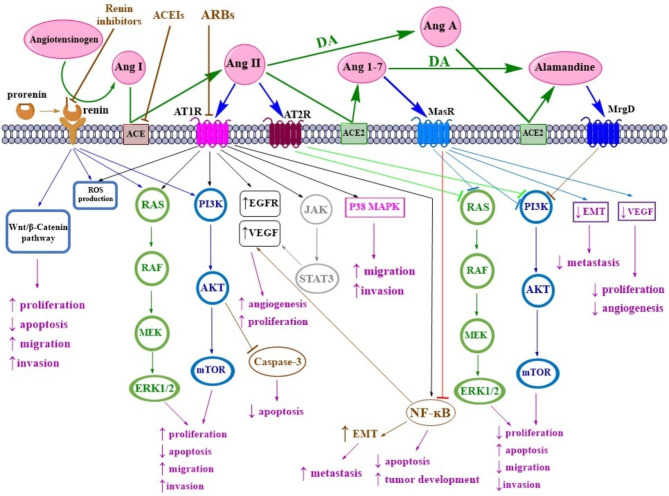
Angiotensin derivatives and their associated receptors The downstream pathways affecting proliferation, apoptosis, angiogenesis, and cancer as well as an overview of the receptors and effectors affecting the RAAS pathway is displayed and the dual effect of this pathway on cancer is depicted. The interactions between Ang II/AT1R and Ang I/AT2R and Ang1-7/Mas and alamandine/MrgD receptors in the RAAS pathway can have a significant impact on the development of cancer. Ang I/AT2R, Ang1-7/Mas, and alamandine/MrgD interactions can have anticancer effects while Ang II/AT1R interactions can be involved in the development of cancer. Activation or inhibition of the PI3K/AKT/mTOR pathway and the RAS/RAF/ERK1/2 pathway, which are downstream of the RASS pathway, are the most effective pathways in cancer development or cancer prevention

The relation between RAAS and cancer has recently been highlighted, and various researches have emphasized the role of RAAS dysregulation in cancer. It is noteworthy that different components of RAAS can have different roles in carcinogenesis [4]. (Fig. 2 displays an overview of different receptors and effectors of the RAAS pathway and its downstream effectors)

Different types of receptors and effectors can affect RAAS regulation. PRR, AT1R, AT2R, and Mas receptor are among the receptors involved in the pathophysiology of RAAS, whose inhibition or activation can impact cancer progression. Among the effectors involved in this pathway, Ang II, Ang 1–7, and Ang 1–9 are of major concern [78].

Endothelial cells and fibroblasts, as well as other major components of the tumor microenvironment, together with tumor cells themselves, could be involved in producing and expressing RAAS components that promote angiogenesis [79]. The creation and secretion of cytokines (TNF, IL-1, and IL-6), growth factors (VEGF), and the production of ROS by neutrophils and macrophages in inflammatory and hypoxic environments are also facilitated by RAAS signaling pathways [80].

### Ang II/AT1R Pathway

Along with the effects of AT1R on the regulation of blood pressure, it can also lead to enhanced proliferation, migration, motility, invasion, angiogenesis, and inhibition of apoptosis [81, 82]. It appears that the AT1R pathway seems to be the basic RAAS pathway that is implicated in tumor development, tumor proliferation, and angiogenesis by increasing the expression of epidermal growth factor receptor (EGFR) or vascular endothelial growth factor (VEGF) [2, 83, 84]. Furthermore, research evidence has revealed that AT1R activation in DU145, LNCaP, and PrSC cells can lead to elevated Janus kinase signal transducers and activators of transcription (JAK-STAT) signaling, mitogen-activated protein kinase (MAPK) activation, and cell proliferation [85, 86]. In fact, AT1R-activated cells recruit nonreceptor tyrosine kinases, for instant Src, that recruit a molecular complex containing JAKs [81]. ATR1 activation can exert antiapoptotic effects in vascular endothelial cells by promoting cell survival and also by inhibiting caspase-3 activity via activating the PI3K/Akt signaling pathway [87, 88]. AT1R upregulation has been found in many different types of cancer, for example, breast, pancreatic, and lung cancers [83].

The involvement of AT1R in tumorgenesis may be explained as part of a complicated process involving a number of cell signaling pathways within endothelial cells, including EGFR and MAPK/ERK1/2 [89, 90, 86, 91]. Ang II can induce VEGF-A expression in endothelial cells through AT1R and ERK1/2 signaling, which are necessary for neovascularization and tumor growth. This promotes neo-angiogenesis in different tumors such as hepatocellular, ovarian, pancreatic, and bladder cancers [92, 93, 94, 95]. Aside from promoting angiogenesis, Ang II/AT1R also has the potential to relate to malignancy via EGFR transactivation. This can hijack downstream signaling pathways associated with malignant transformation and activate molecular cascades that promote cancer cell proliferation [96]. In summary, the PI3K/AKT/mTOR, Jun activating kinase II (JAK)/signal transducer and activator of transcription 3 (STAT3), and RAS/RAF/ERK1/2 pathways are three molecular cascades that can be involved in downstream of the AT1R [96, 97, 98]. The PI3K/AKT/capsase3 and p38MAPK pathways are all activated via VEGF receptors in endothelial cells, which further prevents apoptosis and promotes proliferation [96]. AT1R activation can increase cell proliferation in prostate cancer cell lines and cells generated from prostate stromal cell lines by activating the MAPK and STAT3 signaling pathways [85, 99, 86]. In patients with high blood pressure, overstimulation of AT1R is observed, which can affect vascular remodeling and facilitate cell migration during metastasis [100].

It has been discovered that Ang II can work synergistically with NF-κB in cancer development [101]. The transcription factor NF-κB is expressed in all cell types and is important for metastasis and cancer progression and is considered a survival transcription factor that inhibits apoptosis and increases cancer progression. NF-κB is also capable of controlling metastasis by inducing adhesion molecules which can help the spread of cancerous cells by adhering to vascular endothelial cells and entering the target site [102]. NF-κB can also promote angiogenesis by upregulating VEGF, which promotes neo-angiogenesis throughout migration and invasion [102, 103]. A study demonstrated that Ang II can promote cellular migration as well as induce phosphorylation of PI3K/Akt. Therefore, in general, Ang II has the ability to activate the AT1R/PI3K/Akt pathway, which can ultimately lead to elevated NF-κB and IKKα/β activity. This can increase matrix metalloproteinase, MMP2 and MMP9 expression and cell migration [97]. In breast and gastric cancers, Ang II/AT1R activation can promote survival and invasion through the NF-κB pathway, which is suppressed by the Ang 1–7/MAS receptor [97, 87]. In endothelial cells, NADPH oxidase and NF-κB induce angiogenesis [96]. Therefore, NF-κB activation through Ang II/AT1R signaling enhances tumor development and inhibits apoptosis [104]. It is noteworthy to mention that ACEIs and ARBs might be able to decrease NF-κB activity [105].

Ang II enhances cell growth and proliferation in various ways, such as through affecting tyrosine kinase [106], transforming growth factor-beta (TGF-β) [107], and activating mTOR pathways [106]. Ang II stimulation can increase the proliferation of breast cancer cells, which is regulated through MEK and PI3K signaling [108, 90]. Some studies have suggested that EGFR inhibition is required to fully prevent Ang II-mediated proliferation. In other words, Ang II-mediated proliferation in cancer involves complex mechanisms other than direct signaling from AT1R, such as EGFR transactivation [109, 90, 110]. Ang II can increase ROS production. A direct RAAS activation by Ang II in the tumor microenvironment might result in the production of ROS generation and pro-angiogenic and pro-inflammatory signaling [111]. ROS can function as signaling molecules that help regulate the pathways critical for cytoskeletal contraction control and cell proliferation stimulation. Additionally, the Ang II/AT1R complex activates signaling pathways that regulate the production of extracellular matrix and enhances cell adhesion [112]. It is noteworthy that Ang II proliferation is usually related to certain types of cancer, but there is significant variation in responses even within a cancer type, suggesting that the genetic background of the cancerous cells can influence the cell proliferation potential [111].

Angiotensin-converting enzyme inhibitors (ACEIs) such as Captopril, Enalapril, Lisinopril, etc. can prevent the conversion of Ang I to Ang II by inhibiting the ACE enzyme. Therefore, less Ang II is produced and as a consequence the function of Ang II/AT1R pathway is reduced. Therefore, the use of ACEIs may have a preventive effect in cancer [100]. It should be noted that due to the existing conflicting results, more studies are required to draw definitive conclusions. Angiotensin receptor blockers (ARBs) such as Losartan, Valsartan, Telmisartan, Candesartan, etc. can block AT1 receptors. Therefore, it is possible that ARBs lead to the inhibition of the downstream pathway of this receptor by blocking AT1R and ultimately lead to a decrease in proliferation and cancer prevention. [113, 8]. However it is noteworthy due to conflicting results more studies are needed.

### PRR pathway

It is well recognized that PRR is an important receptor involved in the regulation of RAAS. PRR can be involved in PI3K/AKT/mTOR, MAPK/ERK, and Wnt/β-catenin pathways, thus it can play a role in different pathological and physiological conditions, such as cancer [49]. Up-regulation of PRR has been found in some types of cancers for instance pancreas cancer [56], prostate cancer [114], and leukemia [115]. It has been found that PRR can activate MAPK/ERK as well as PI3K/AKT/mTOR signaling pathways [116]. In pancreatic cancer cells, PRR pathway silencing reduces the expression of AKT, ERK1/2, and NF-κB [56]. In different cell types such as mesangial cells, monocytes, neurons, and collecting duct cells, PRR can activate MAPK/ERK signaling and up-regulate ERK1/2 [117]. ERK1/2 activation thus promoting cellular proliferation and inducing TGF-β generation, which plays a role in the pathogenesis and metastasis [118, 119]. PRR can also induce ROS production in an Ang II-independent manner and thus can contribute to PI3K/AKT/mTOR and MAPK/ERK signal transduction [120]. It is generally recognized that the Wnt/β-catenin pathway is involved in the progress of a variety of cancers, including breast [121], gastric [122], prostate [123], colorectal [124], and adrenocortical cancers [125]. PRR can be considered a crucial part of the Wnt receptor complex that can act as an adapter protein and therefore facilitate the attachment of Wnt ligands to the Wnt receptor complex [52]. Further research demonstrated that PRR probably can promote colorectal [58], pancreatic [55], and brain [54] cancers through the Wnt/-catenin signaling pathway. These data collectively suggest that PRR probably exerts its cancer-promoting effects through the PI3K/AKT/mTOR, the MAPK/ERK, and the Wnt/β-catenin pathways [116]. Renin-inhibitors can exert their anticancer effects through decreasing the plasma renin activity by fulfilling its active sites and preventing the conversion of Angiotensinogen to Ang I [126].

### Mas receptor and the AT2R pathways

Components of RAAS can have different roles in carcinogenesis. The signaling pathways triggered by the Ang II/AT2R signaling are poorly understood and recognized, compared with those.

triggered by Ang II/AT1R signaling, however generally, it has been demonstrated that activation of AT2R has protective effects, in contrast to AT1R [127, 81]. Therefore, while AT1R has proliferative properties, AT2R and Mas receptor have shown anti-proliferative effects [128, 129]. Ang 1–7 suppresses angiogenesis along with cell proliferation contrary to Ang II [130, 131]. The Ang 1–7/Mas receptor and the Ang II/AT2R pathways are believed to be antagonistic to several cellular functions of the Ang II/AT1R pathway [79].

AT2R can inhibit cell proliferation while it can stimulate apoptosis in different types of cells, such as lung cells, vascular smooth muscle cells, endothelial cells, cardiomyocytes, and prostate cancer [132, 133, 134, 135]. Additionally, AT2R overexpression can cause apoptosis without the involvement of Ang II via the activation of caspase 3, caspase 8, and p38 MAPK, which is regulated by an extrinsic cell death-signaling pathway that is partly dependent on p53 [136, 137]. The major signaling pathways that AT2R activates include the PLA2/arachidonic acid signaling, the NO-cGMP system, and the activation of protein phosphatases. There are three different classes of protein phosphatases that are significantly activated by the Ang II/AT2R complex which consist of SH2 domain-containing phosphatase (SHP-1), protein phosphatase 2 A (PP2A), and MAP kinase phosphatase 1 (MKP-1). Therefore, these proteins can regulate the inhibition of the PI3K/AKT, ERK/MAPK, and JAK phosphorylation cascades [81]. It has been discovered that AT2R signaling can inhibit cell survival, cell proliferation, invasion, and migration by inhibiting the MEK1/2 and Ras proteins and MAPK pathways, which is facilitated by phosphatase-mediated dephosphorylation events [138, 139].

Mas receptor interference by Ang 1–7 can exert antiproliferative, anti-inflammatory, anti-migratory, and anti-fibrotic effects. It can affect vascular and cellular growth mechanisms and exert inhibitory effects [140] as well as preventing the growth of tumor cells in various cancers like esophageal squamous cell carcinoma [141], nasopharyngeal carcinoma [142], lung cancer [143], and prostate cancer [144]. Ang1-7/Mas axis can exert its beneficial effects on cancer through various mechanisms, such as exerting inhibitory effects on cellular proliferation and preventing metastasis and invasion [145]. In cultured tumor cells, the anti-proliferative Mas receptor/Ang 1–7 axis is probably mediated by the inhibition of ERK signaling [146]. In human nasopharyngeal carcinoma xenografts, it was reported that Ang 1–7 can down-regulate PI3K/Akt/mTOR pathway and inhibit tumor growth via autophagy [147]. A similar effect was reported in breast cancer as well, where Ang 1–7 reduced activation of the PI3K/AKT pathway, and VEGF expression [148].

AT2R receptor-interacting proteins (ATIP) are proteins that can interfere with the balance of AT1R and AT2R. The first ATIP member to be identified as constitutively interacting with AT2R at the cellular membrane is ATIP-1. The inhibitory effect of AT2R on ERK phosphorylation, receptor tyrosine kinase activation, and cell proliferation is predominantly mediated by ATIP-1 [149]. Additionally, ATIP-1 is involved in the transport and signaling of AT2R receptors. Another protein with tumor-suppressing properties that interacts with AT2R is ATIP-3. ATIP-3 levels is found to be lower in invasive breast cancer, and also the proliferation of tumor cells is decreased when ATIP3 expression is restored in breast cancer cells [150].

The endogenous substance alamandine exists in the blood circulation and cardiovascular system. The biological activities of alamandine are mediated via MrgD and are similar to those of Ang 1–7, including antihypertensive, antifibrotic, vasodilation, and central cardiovascular effects [151]. Alamandine can also reduce pulmonary fibrosis through the MrgD receptor. Studies have shown that alamandine/MrgD receptor axis appears to be critical for defending the body from SARS-CoV-2 disease [152]. Studies have revealed that alamandine/MrgD signaling, which is currently poorly understood, may be involved in the activation of different signaling cascades, such as the c-Src/p38 MAPK pathway [153]. Alamandine/MrgD signaling can also activate protein kinase A and protein kinase C signaling pathways [154, 155]. Additionally, it has been reported that alamandine can activate the AMPK pathway [156], inhibit the MAPK pathway [157], and activate Jun-N-terminal kinase (JNK) phosphorylation to inhibit the NF-κB pathway [158]. Signaling cascades triggered by alamandine/MrgD activation may mediate almandine protective functions and counteract Ang II’s detrimental effects [81]. Further studies on the physiological effects of the alamandine/MrgD seem necessary and can be helpful in better understanding of the downstream of this pathway and the effects it probably has beyond the cardiovascular system effects, such as possible anti-proliferative effect, anti-cancer effect, etc. It has been reported that alamandine can induce a shift in anaerobic metabolism to aerobic metabolism in tumor cells, negatively regulate PI3K/AKT/mTOR signaling pathway, and activate FoxO1 transcriptional factor. These events may account for the observed anti-proliferative effects of alamandine, at least partially [159].

## Drugs inhibiting RAAS elements

ACEIs and ARBs are the two primary RAAS inhibitors. These two are usually used in renal and cardiovascular disease management [160, 161, 162]. While ARBs specifically prevent Ang II from binding to downstream angiotensin receptors, ACEIs are able to inhibit the synthesis of Ang II [163]. Studies have indicated that ACEI/ARBs can alleviate cancer [83]. Since down-regulating RAAS has inhibitory effects on metastasis, cancer growth, and angiogenesis, ACEI and ARBs have been proposed as possible treatment options for cancer [164]. Following the effect of ARBs, the ability of Ang II for binding to AT1R is diminished, limiting proliferation capacity [78, 128]. The favorable effects of ARBs on the development of cancer may be described by the dominance of Ang 1–7 and AT2R over Ang II and AT1R. A study conducted on breast cancer showed that Ang 1–7 level has decreased, and the ACE2/Ang 1–7/Mas pathway has been down-regulated [165, 166]. Researchers have discovered that the common ARB medicine, losartan, plays a role in lowering tumor growth rates in human pancreatic, breast, and prostate cancer cell xenografts [77, 167, 168]. Similarly to this, captopril, which belongs to the ACEI drugs group decreased the extent of liver metastases in addition to inhibiting tumor angiogenesis [169]. According to a study conducted on prostate cancer, ARBs such as telmisartan and candesartan have been able to suppress the expression of AT1R, induce apoptosis, and suppress cell proliferation [170, 171, 172]. A study showed that individuals with rectal cancer who consumed ARBs or ACEIs responded better to neoadjuvant therapy [173]. The use of RAAS inhibitors greatly enhanced overall survival rates and patient outcomes in different types of cancers, according to several other trials [4, 174]. (Some of the studies conducted by ACEI drugs and ARBs are listed in Table 1.)

**Table 1:** Effect of RAAS inhibitors and their associated targets in various cancers

Type of cancer	Drug	Target receptors	Clinical study	Outcome	Reference
**Ovarian cancer**		Ang II/AT1R	Human epithelial ovarian cancer tissues	In ovarian cancer patients, AT1R could be associated with tumor angiogenesis and a poor prognosis.	[339]
	Candesartan and PD123319	AT1R	- Human epithelial ovarian tumor tissues - SKOV-3 and HRA cell line - BALB/c mouse model of peritoneal carcinomatosis	- Ovarian cancer expresses AT1R, which is involved in angiogenesis and tumor growth. - A promising approach to treating ovarian cancer may be AT1R blocking therapy.	[95]
	losartan and captopril	PAX8	- OVCAR-4, OVCAR-8 and HEK293T cell lines	- Losartan and captopril can inhibit PAX8 expression and function. Therefore, they may be a suitable pharmacological target for various forms of HGSC.	[179]
	losartan	Ang II and AT1R and MCS formation	- A2780 and Ovca429 cell lines - The isogenic non-metastatic (NM) and highly metastatic (HM) cells - NOD-SCID mouse	- Ang II increases MCS formation and rise peritoneal metastasis of epithelial ovarian cancer cells. - By overexpression of SCD1, AT1R activation enhances lipid desaturation, which finally can lessen endoplasmic reticulum stress in MCS.	[183]
		sPRR	− 197 primary epithelial ovarian cancer patients	- sPRR probably doesn’t have prognostic, predictive, or diagnostic value in epithelial ovarian cancer	[176]
		Ang 1–7, Ang 1–9 and Ang 3–7	- OVPA8 cell line	- Peptides from RAS have the ability to regulate how nutrient-deprived tumors adapt to starvation. - The availability of growth factors and nutrition has a significant impact on how angiotensin peptides affect cancer cells.	[340]
		ACE	41epithelial ovarian cancer patients and 19 healthy controls	- serum ACE levels has been increased - circulating ACE is probably related to pathobiologic process	[177]
	losartan and CGP42112A	AT1R and AT2R	− 58 human epithelial ovarian cancer tissues − 2774, A2780, PA-1, SKOV3ip1, HeyA8 cell lines - Human umbilical vein endothelial cells (HUVEC)	Dual regulation of AT1R and AT2R, which inhibits angiogenesis and cancer cell survival, might be a potential for treating epithelial ovarian carcinoma.	[178]
	losartan	AT1R	- SKOV3ip1 and Hey-A8 ovarian cancer cells - orthotopic human ovarian carcinoma xenograft models - mouse fibroblast (10T1/2) - mice (*Mus musculus*), and rats (*Rattus norvegcus*)	losartan therapy can improve paclitaxel’s effectiveness losartan can increase medication delivery and improve vessel perfusion. losartan it can lower both the incidence and the volume of ascites produced.	[184]
**Prostate cancer**		Ang II and relaxin 2	- PNT1A cells	The development of some malignancies, particularly prostate cancer, may be influenced by the dysregulation of locally released peptide hormones including Ang II and relaxin 2.	[189]
	recombinant adenoviruses encoding AT2R (Ad5-CMV-AT2R-EGFP)	AT1R and AT2R	- DU145 cell lines - BALB/c nude mice	The findings demonstrated that AT2R overexpression can prevent prostate cancer tumor growth in vivo by preventing tumor cell proliferation and triggering apoptosis. The gene AT2R has the potential to be helpful in prostate gene therapy.	[185]
	an AT2R agonist (C21)	AT2R	- LNCaP cells - transgenic rat for adenocarcinoma of prostate (TRAP) model	A potential new chemo preventive drug against human prostate cancer is AT2R agonist.	[186]
	fimasartan, losartan, eprosartan and valsartan	AT1R	PC-3, DU-145 and LNCapLN3 cell lines	Prostate cancer cells may exhibit anti-metastatic action and autophagy-associated cell death when exposed to ARBs.	[189]
	candesartan	AT1R and Caveolin-1	- Metastatic prostate adenocarcinoma cells (PC-3)	- The mRNA and protein levels of caveolin expression may be regulated by candesartan. - growth inhibition of metastatic PC3 cell line	[341]
	olmesartan	AT1R, ACE and Ang I/II	- DU145 and LNCaP cells - human prostate cancer cell lines - prostate stromal cells (PrSCs)	Prostatic RAS is overexpressed in hormone-resistant prostate cancer tissue, and different types of hormonal stimulation affect the expression of its components.	[342]
	Ang 1–7	Ang 1–7	- Human PC3 prostate cancer cells - SCID mice	For advanced prostate cancer, Ang 1–7 may function as an anti-metastatic agent and anti-angiogenic.	[343]
	Ang 1–7	Ang 1–7	- LNCaP human prostate cancer cells - Male athymic mice	A potential anti-angiogenic therapy for prostate cancer may be Ang 1–7.	[344]
	Ang 1–9 and Ang 3–7	AT1R AT2R AT4R Mas receptor	PNT1A cell line	Ang1-9 and Ang3-7 have opposite effects Ang1-9: cause cell divisions, increase cell motility, and stimulate the expression of genes including VEGF, HIF-1, VIM, and REL Ang3-7: exhibit no mitogenic action	[193]
	Ang 1–9 and Ang 3–7	AT1R AT2R AT4R Mas receptor	LNCaP cell line PC3 cell line	Ang 1–9 and Ang 3–7 might alter the overall number of steroid receptors in aggressive prostate cells. the switch from hormone-dependent prostate cancer to hormone-refractory illness may be facilitated by Ang 1–9 and Ang 3–7	[194]
**Breast cancer**	Candesartan	AT1R	Xenograft mice model	Inhibition of tumor growth and angiogenesis	[345]
	Ibesartan	AT1R	BC cell line	Suppression of cell proliferation	[346]
	Olmesartan	AT1R	BC cell line	Apoptosis induction	[347]
	Losartan	AT1R	Mouse model	Suppression of tumor cell proliferation, Reduction of inflammatory cytokines.	[348]
			BC Cell line	Reverse the Ang II-induced cancer cell proliferation and VGEF-A upregulation, Reduction in cell adhesion and invasion	[198, 345]
			4T1 and EMT6 murine TNBC cell lines	Improves the Antitumor Efficacy of Dox-L Chemotherapy, Combination Therapy Involved α-PD1 Immunotherapy Alleviates Primary Tumor Burden and Lung Metastases	[240]
			mouse (E0771 and 4T1 models)	Reduces stromal fibrosis, signalling Reduced solid stress, increase blood perfusion in tumours, enhance drug and oxygen delivery.	[77]
	Captopril	ACE1	TAM resistant BC cell line	Prevention of TAM resistant cells	[349]
			BC cell line with adipocyte-CM exposure	Reduction of cancer growth and inflammation in TME	[350]
			BC cell line	Inhibition of TF and VEGF expression	[351]
	Perindopril	ACE1	Mouse tumor xenograft and corneal micropocket model	Inhibition of tumor growth and angiogenesis	[352]
	ACEI and / or ARBs	ACE 1and / or AT1R	Human studies (Meta-Analysis)	Decreased breast cancer risk with long-term ACEI /ARB use	[353]
			Human studies: Retrospective study	No association with ACEI /ARB use and with disease free or overall survival	[242]
				higher incidence of invasive lobular carcinoma subtype	[354]
**CRC**	Telmisartan	AT1	In vitro	Inhibit cell proliferation, G0/G1 cell cycle arrest,	[254]
	Captopril	ACE	Mouse Model	Reduce EMT and invasive phenotype, Reduced tumor growth, reduction in volume of colorectal cancer liver metastases	[169, 355]
	Ibesartan	AT1R	Mouse Model	Reduced tumor growth, reduction in volume of colorectal cancer liver metastases	[169]
	Candesartan	AT1R	In vitro / CRC mouse models	Inhibition of CRC cell growth and migration, enhanced necrosis in CRC cells and tumors	[356]
	Valsartan	AT1R	In vitro/CRC mouse models	Inhibition of tumor cell growth, Induction of apoptosis, Inhibition of cell migration, ameliorate inflammation	[357]
	Losartan	AT1R	Xenograft Model	Inhibition of tumor growth, migration and angiogenesis, increase pro-inflammatory cytokines	[358]
	LP2	AT2R	Xenograft model	Apoptosis in patient-derived xenografts of colorectal carcinoma, synergism demonstrated in combination of LP2 with 5-FU and the EGFR inhibitor erlotinib	[251]
	ACEis/ARBs	ACE1/AT1R	Human studies: Retrospective cohort study	association with a lower colorectal cancer risk in a duration-response manner	[359]
			Human studies: Cohort analysis of SEER-Medicare database	ACE inhibitors associated with decreased mortality	[257]
			Human studies : Retrospective study	ACEI/ARB + BB exposure was associated with decreased mortality compared to unexposed individuals, decrease in cancer progression, decrease cancer-related hospitalizations	[259]
				ACEI/ARB treatment may reduce tumor recurrence in left-sided CRC and early-stage CRC.	[260]
**Gastric cancer**	CandesartanTelmisartan	AT1R	Human GC cell line	Reduce cellular proliferation	[266]
	Candesartan		Xenograft Model	Inhibition of Tumor growth, Suppression of EMT and fibrosis	
	TCV-116	AT1R	Xenograft Model	Inhibition of tumor growth, reduce VEGF	[360]
	Olmesartan	AT1R	N87 and MKN45 cell lines (Human GC cell line)	significant decrease in invasive ability of cancer cells	[263]
	RAAS inhibitors	ACE1/AT1R	Human studies: Cohort studies	Lower incidence of GC rate	[361]
**Pancreatic cancer**	Losartan	AT1R	Orthotopic PDAC in mice	Increase drug uptake and functional microvasculature, Inhibition of Cell proliferation and VEGF expression, improve survival rate	[362, 363]
			A Phase 2 Clinical Trial (Total Neoadjuvant Therapy With FOLFIRINOX in Combination With Losartan Followed by Chemoradiotherapy for Locally Advanced Pancreatic Cancer)	Prolonged progression-free and overall survival.	[243]
	Captopril	ACE1	In vitro and In vivo studies	Targeted delivery of captopril to the CAFs, significantly down-regulated the deposition of ECM by blocking the TGF-β1-Smad2 and consequently enhance the liposome-encapsulated chemotherapeutic agent gemcitabine.	[364]
	Irbesartan	AT1R	In vitro and In vivo studies	effectively reduced chemoresistance in PDAC	[292]
	Telmisartan	AT1	In vitro	Inhibit the human PDAC cell pro;iferation by cell cycle arrest, The miRNA expresseion profile was markedly altered	[365]
			Orthotopic PDAC mice model	increased vascular perfusion and reduced hypoxia,	[366]
			Wistar Bonn/Kobori (WBN/Kob) rat	Prevented the development of chronic pancreatitis and fibrosis.	[276]
			Human studies: Retrospective analysis	longer overall survival in PDAC patients with non-metastatic disease and resected PDAC patients, improve clinical outcomes in patients with advanced pancreatic cancer in combination with Gemcitabine	[367–369]
			A population study	Improve survival	[293, 370]
**Hepatic cancer**	Candesartan	AT1R	Hepatic carcinoma cell line	Inhibition of proliferation	[306]
	Telmisartan	AT1R	non-alcoholic steatohepatitis (NASH) rats	Prevent hepatocarcinogenesis through inhibition of angiogenesis	[371]
	Irbesartan	AT1R	Xonegraft model	Inhibition of HCC growth and Metastasis, Weaken adhesion to endothelial cells	[311]
	Losartan	AT1R	diethylnitrosamine-induced hepatocellular carcinoma in mice	Decrease cell migration, Metastasis, Inhibition of Neovascularization	[372]
	Fosinopril	ACE			
	Perindopril	ACE			
	Azilsartan	AT1R	hepatocellular adenocarcinoma cell line	Induce cell apoptosis	[373]
	Losartan	AT1R	Mouse model of HCC	reduced liver and peritumoral fibrosis , lenhance anti-PD-1-triggered HCC regression in	[374]
**Esophageal cancer**	Telmisartan	AT1R	EAC Cell lines	Inhibition of proliferation and cell cycle arrest	[332]
			Xenograft Model	Inhibition of tumor growth	
	Benazepril	ACE	Xenograft Model	Inhibition of tumor growth and angiogenesis	[335]
	ACEi and/ or ARBs	ACE/AT1R	Human studies: a nested case control study	Decrease the risk of ESCC and EAC in high daily doses	[375]

## Sex Hormone-dependent Cancers

### RAAS components in ovarian cancer

The high mortality rate of ovarian cancer is due to its nonspecific symptoms. [175, 175] Despite greater knowledge of ovarian cancer, trends in treatment and survival have not changed considerably because it is still difficult to make an early diagnosis [175].

Angiogenesis is related to tumor invasion. AT1R has recently been determined to be highly and effectively expressed in ovarian cancer cells [176] and has been demonstrated that Ang II increases tumor cell invasion and VEGF expression through AT1R. In a mouse model, an AT1R blocker also inhibited cancer angiogenesis and prevented peritoneal dissemination of ovarian cancer [95]. Patients with positive AT1R staining exhibited considerably overall lower survival and reduced progression-free survival when compared to patients with negative AT1R staining. These findings demonstrate that in ovarian cancer, AT1R expression correlates with tumor angiogenesis and a poor prognosis, supporting the idea that AT1R could be considered a crucial molecule in tumor angiogenesis. This demonstrates that AT1R blockade therapy can probably be potentially a new molecular target for ovarian cancer therapy [176, 95]. According to a study that aims to evaluate the probable relationship between circulating ACE enzyme levels and ovarian cancer, it has been determined that ovarian cancer patients have increased serum ACE levels, and circulating ACE may be related to a pathobiological process in the carcinogenesis of ovarian. Therefore, ovarian cancer treatment in the future may involve targeting the RAAS pathway [177]. Dual targeting of AT1R and AT2R has also been investigated in epithelial ovarian carcinoma. It has been demonstrated that a novel therapeutic approach for epithelial ovarian carcinoma may involve dual regulation of AT1R and AT2R, which prevents angiogenesis and cancer cell survival [178].

Paired box transcription factor 8 (PAX8) is one of the most commonly used biomarkers in High-Grade Serous Ovarian Cancer (HGSC). It is expressed in about 90% of patients with HGSC. Although PAX8 is not expressed by the ovarian surface epithelium it is expressed by animal models of HGSC that are derived from it. It is indicated that it could be an effective factor in the development of cancer in addition to being a hallmark of Müllerian origin. PAX8 deficiency in HGSC cells can cause cellular tumor death and decreases invasion and cell migration. PAX8 deficiency can also decrease the amount of TGF-beta secretion, which is a cytokine that is crucial for the modification of the tumor microenvironment. Therefore, PAX8 inhibitors may be a suitable pharmacological target for various forms of HGSC. Losartan and Captopril are both RAAS pathway inhibitors that can prevent PAX8 expression and activity. Therefore, Losartan and Captopril can have beneficial effects on the treatment of ovarian cancer [179]. Losartan and Captopril were identified as drugs that can constantly suppress the expression of the PAX8 protein and inhibit the function of the PAX8 promoter. PAX8 can promote invasion and tumor cell migration through modulating FOXM1 and PKCα expression [180]. In OVCAR8 cells, Captopril and Losartan both inhibited the expression of fibronectin, PAX8, and PAX8 downstream proteins such as PKCα and FOXM1 [179].

The prognostic and predictive efficacy of soluble PRR as a biomarker for clinicopathological outcomes in primary epithelial ovarian cancer patients has been investigated. However, no correlation between levels of sPRR with clinicopathological factors and prognostic data was observed. sPRR seems to have no diagnostic, prognostic, or predictive value in epithelial ovarian cancer [176].

In ovarian cancer, multicellular spheroids (MCS) can be formed, which can lead to disseminating cells to accumulate together and ultimately promote cancer metastasis and prevent cell death [181]. In contrast to the majority of solid tumors that spread through the blood, ovarian cancer typically disseminates through the peritoneal cavity [182]. It has been found that Ang II/AT1R signaling is probably effective in the progress and promotion of ovarian cancer, so it could be considered a critical therapeutic target [183]. Ovarian tissue expresses RAAS components locally, and it is likely that enhanced expression of its components, such as Ang II and AT1R, can be related to the progression of ovarian cancer [177]. Ovarian cancer cells can overexpress Ang II as well as stimulate AGT gene expression to generate more Ang II, which can ultimately result in cancer development and MCS formation [183]. Through lipid homeostasis and stimulation of the lipid desaturation pathway, cancer cells can decrease endoplasmic reticulum stress brought on by saturated fatty acid overload, which ultimately prevents cancer cells from going into apoptosis. Sterol Regulatory Element-Binding Protein (SREBP) is a signaling pathway that Ang II/AT1R signaling can activate to induce lipogenesis. Stearoyl-CoA Desaturase-1 (SCD1) is an endoplasmic reticulum enzyme that is in charge of lipid desaturation. It is one of the lipogenesis enzymes that are upregulated by the SREBP pathway. Therefore, SCD1 can help cancer cells to prevent cell death [183]. AT1R activation can increase lipid desaturation through up-regulation of SCD1, which finally can decrease the endoplasmic reticulum stress of MCS. The above mechanism can clarify the link between elevated levels of AT1R and worse clinical outcomes in ovarian cancer patients [183].

Tumor fibrosis and Ang-driven fibrogenic signaling have been demonstrated to negatively correlate with survival in ovarian cancer patients. A study aimed to improve drug delivery and treatment efficacy by remodeling the dense extracellular matrix in two orthotopic human ovarian cancer xenograft models. It has been discovered that by using losartan to target the Ang signaling axis, extracellular matrix content, and related “solid stress” can be decreased, improving the anticancer therapeutic impact. It was also found that losartan therapy can improve paclitaxel’s effectiveness by improving the tumor microenvironment. Consequently, it may lead to increased medication delivery and improved vessel perfusion. Although losartan medication alone does not diminish tumor burden, it can lower both the incidence and the volume of ascites produced. These results offer the justification and supporting information for a clinical investigation on losartan and chemotherapy combination therapy for ovarian cancer patients [184].

### RAAS components in prostate cancer

Metastasis occurs in a significant percentage of prostate cancer cases. Most patients eventually develop chemo resistance and lose their responsiveness to additional hormonal therapy. Therefore, the development of novel therapeutic strategies for the treatment of prostate cancer is necessary [185].

AT1R and AT2R receptors can be considered possible therapeutic targets for the treatment of prostate cancer. AT1R inhibition and AT2R activation can have beneficial effects on prostate cancer treatment. Investigation on the effects of AT2R stimulation revealed that in vivo and in vitro treatment of an AT2R agonist increases apoptosis and reduces androgen receptors expression, hence lowering cell proliferation. Thus, AT2R activators were recommended as potential chemo-preventive therapy candidates for human prostate cancer [186]. Early stages of prostate cancer were shown to have an overexpression of Ang II receptors [187]. Also, there is an association between AT1R expression and prostate cancer cells’ potential for metastasis [188]. AT2R overexpression in prostate cancer can reduce tumor growth, decrease Ki-67 expression, and induce apoptosis, according to an in vivo investigation. Furthermore, there was an inverse relationship between AT2R expression and prostate cancer aggressiveness [185].

RAAS was previously found to express itself in both healthy prostate tissue and cancer cells, demonstrating its importance in both normal cell function and tumor cell transformation. In healthy prostate cells, RAAS can be involved in sperm motility, spermiogenesis, and sperm survival. Long-term exposure of healthy prostate cells to Ang II can alter cellular morphology, and increase cell survival and cell proliferation via upregulating BCL2/BAX ratios. It can also increase the degradation of the extracellular matrix. In summary, Ang II expression dysregulation in the prostate can increase the risk of prostate cancer [189].

It is reported that prolonged administration of ARBs or ACEIs in hypertensive patients can reduce the incidence of prostate cancer [190]. Moreover, consumption of high concentrations (100–400µM) of ARBs has been reported to enhance the death of prostate cancer cells via increasing cancer cell autophagy and increasing the expression of autophagy-related genes. In addition to the advantages of ARBs, Fimasartan was capable of reducing prostate cancer migration; as a result, it might be a potential treatment agent for prostate cancer in hypertensive patients [191].

Protein Tyrosine Kinase (PTK) activity was reported to be decreased by Ang 1–7 and Ang II/AT2R in androgen-independent prostate cancer. PTKs were shown to be elevated in the advanced stages of prostate cancer. This suggests that Ang 1–7 and Ang II may be involved in providing protection against prostate cancer in advanced stages, or in androgen-independent prostate cancer [192].

In a study, the effects of Ang 1–9 and Ang 3–7 in prostate epithelial cells have been investigated. The findings of this study demonstrate that Ang 1–9 and Ang 3–7 have opposite impacts on the in vitro biological characteristics of prostate cells. By causing cell divisions, increasing cell motility, and stimulating the expression of genes including vascular endothelial growth factor (VEGF), hypoxia-inducible factors (HIF-1), vimentin (VIM), and REL proto-oncogene, NF-kB subunit (REL), Ang1-9 appears to have pro-cancer effects. Ang3-7, on the other hand, exhibited no mitogenic action [193]. Further investigation on the effects of Ang 1–9 and Ang 3–7 on prostate cancer was also conducted. The results imply that Ang 1–9 and Ang 3–7 can affect the biological characteristics of prostate cancer cells via modulating genes involved in the inflammatory and steroidogenesis pathways. Additionally, it was demonstrated that Ang 1–9 and Ang 3–7 might alter the overall number of steroid receptors in aggressive prostate cells. This indicates that the switch from hormone-dependent prostate cancer to hormone-refractory illness may be facilitated by these peptides [194, 186]

### RAAS components in breast cancer

RAAS components are presented in both normal and cancerous breast tissues. The physiological activities of local RAAS, particularly in the epithelial tissue are tissue growth and modeling [195–197]. Much evidence suggests that dysregulation of this system influences cancer progression, metastasis, and resistance to cancer therapeutics [198, 199]. Various components of RAAS play different roles in the biology of breast cancer which are discussed in the following sections.

#### AT1R pathway

The Ang ΙΙ/AT1 receptor axis as the major and most well-known RAAS pathway plays a pivotal role in many cellular processes including proliferation, migration, angiogenesis, and inflammation, and is strongly associated with tumorigenesis [200]. AT1R activation through binding of Ang ΙI, induces cell proliferation and attenuates cell death via PI3K/AKT signaling and ERK/MAPK signaling pathways [201]. Furthermore, PI3K/AKT activation by AT1R influences NF-κB which is involved in cell migration [97]. It also causes angiogenesis by increasing the VEGF generation to maintain tumor growth [79, 202]. In the triple-negative breast cancer cell line, RAAS plays a role in angiogenesis by increasing HIF-2α, and TIMP-1 [203]. RAAS activation through the AT1 receptor is involved in migration and metastasis through up-regulation of MMP-2 and MMP-9 expression and enzymatic activity in breast cancer cells [204]. It has also been proven that over-expression of Intracellular Adhesion Molecule-1(ICAM-1) which is associated with the aggressive phenotype of breast cancer cells results in response to Ang II treatment [205].

A study by Ma et al. proposed that the role of AT1R for lymph node metastasis is AT1R-mediated and is through CXCR4/SDF-1α. The study has revealed that this signaling pathway would further activate the members of the FAK family and upregulate Rho A. The FAK/RhoA signaling stimulates the ROCK, which promotes cell contractility and migration to reach lymph nodes [206].

Inflammation, another mechanism of AT1R activation, is viewed as a hallmark-facilitating characteristic of cancer [207]. The tumor microenvironment simulates an inflammation site that possesses remarkable advantages for tumor progression, metastasis, and immunosuppression [208, 209]. There is strong evidence that could confirm that RAAS activation via AT1 receptor signaling may promote an inflammatory TME [210–212]. Downstream activation of the AT1 receptor in breast cancer cells, triggers CBM/NF-κB-mediated inflammatory response by releasing inflammatory cytokines and growth factors including IL-1β, IL-8, IL6, and VEGF to the microenvironment [213, 214]. Another possible relationship between Ang signaling via the AT1 and TME that can be addressed is Ang II–AT1R-mediated activation of NADPH oxidase (NOX) complexes leading to ROS generation and oxidative stress [112, 207, 215–217] which is involved in inflammation and angiogenesis in TME [111]. Immune and stromal cellsin TME also contain immune cells and stromal cells that are capable of expression of RAAS components [218–221]. Tumor-associated macrophages (TAMs) are the most abundant immune cells that reside in breast tumors [222]. These intratumoral immune cells contribute to the formation of immunosuppressive microenvironment [223, 224]. Ang II induces TAM infiltration into TME. In a study by Nakumara et al.the abrogation of Ang II effect via AT1R by receptor blockade or AGT (Angiotensinogen)-silenced breast tumor cells decreased the infiltration of macrophage and fibroblasts into tumor regions and further improved tumor response to immune-checkpoint inhibitors such as programmed cell death protein 1 (anti-PD-1) [143, 199, 225].

#### AT2R pathway

There is not much known about the role of the AT2 receptor in breast cancer. AT2 and AT1 receptor co-expression in breast tumors on one hand and the fact that both of these receptors are the target of the local Ang II on the other hand, suggests the interplay roles of these receptors [226–228]. AT2 receptor is expressed at low levels in normal breast ducts, however; its expression significantly increases under pathological conditions such as breast hyperplasia, Ductal Carcinoma In Situ (DCIS), and aggressive breast cancer [229]. On the other hand, studies report that there is no correlation between the expression of AT2R and clinical and pathological characteristics in breast cancer including proliferation and angiogenesis [226]. Another study revealed the possible role of AT2 receptor activation in migration and metastasis inhibition in breast cancer cells by shutting down CAV1/Rab5/Rac-1 signaling pathway [138].

#### ACE2/Ang 1–7/ mas receptor pathway

ACE2/Ang 1–7/ Mas Receptor is another arm in the RAAS that is recognized with the counter-regulatory role for Ang II/AT1R [230]. It is demonstrated that Ang 1–7, counteract Ang II-induced metastatic functions in breast cancer cells. Accordingly, VEGF and MMP-9 expression and activation are abolished by Ang 1–7 effect through Mas receptor signaling. It is further indicated that Ang II-induced ERK1/2 activation which also promotes EMT in cancer is inhibited by Ang 1–7 [231]. Additionally, down-regulation of ACE2/Ang 1–7/MasR, induce metastasis by activating the SOCE (Store-operated calcium Entry) and PAK/NF-κB/Snail1 pathways [165]. The role of ACE2 as a potential antitumor component in the RAAS family has been reported in breast cancer. It is suggested that ACE2 inhibits angiogenesis by suppressing the VEGFa/VEGFR2/ERK pathway in breast cancer [232]. A pan-cancer analysis studied the correlation of immunological features of ACE2 in breast cancer. The study uncovered that ACE2 expression in high levels is associated with an inflamed TME or so-called immune-hot tumors. ACE2 expression is positively related to the levels of immunomodulators and infiltrating levels of TIICs (Tumor-Infiltrating Immune Cells) in breast cancer TME. In fact, it is suggested that the product of ACE2, Ang 1–7 exhibits anti-tumor effects by remodeling TME and sensitizes the tumor to respond to chemotherapy and anti PD-1 immunotherapy [233]. Another study demonstrated that in breast cancer cells the production of Ang 1–7 is significantly lower in comparison to healthy breast tissue, whereas the expression of the Mas receptor was a few times higher than in normal cells [234]. Considering the multiple benefits of anti-cancer effects and the protective functions of Ang 1–7/MasR signaling, the suggestion of balancing Mas receptor activity with agonist substances may be a potential therapeutic option in breast cancer [235].

In clinical perspective, the question of inhibiting the RAAS by current ARBs and ACEIs as a therapeutic potential target in breast cancer still remains a topic of debate. There is a growing body of evidence in pre-clinical and clinical studies which shows the positive role of RAAS inhibitors in cancer treatment intervention [236] One reason that make RAAS an attractive candidate as a therapeutic strategy for treatment of breast cancer, is the desmoplastic reaction in breast tumors. Several studies demonstrated that RAAS inhibitors can normalize the fibrotic stroma in cancer [237]. In a study by Chaugan et al. data indicated that Losartan, is a dual inhibitor of collagen and hyaluronan synthesis. The data suggested that losartan decompress vessels in desmoplastic tumors by reducing solid stress and antimatrix effects. This effect further enhance the drug delivery and effectiveness of chemotherapy. [77, 238–240].

A recent meta-analysis by Xie et al., which emphasized on the association between RAAS inhibitors and breast cancer demonstrated that long-term use of RAS inhibitors, reduces the risk of breast cancer compared with non-users [241]. However the results of another meta-analysis did not show association with disease free and Overall survival [242]. The heterogeneity in terms of tumor stage, the hormone receptor status and other tumor characteristics may affect the data analysis to report contrary results [237]. Nonetheless, there are clinical trials on different types of cancer including breast cancer to explore the potential role of RAAS inhibitors as adjunctive treatment options in breast cancer [243].

### RAAS Components in Colorectal Cancer (CRC)

RAAS receptors and mediators have been identified in normal colon tissue. Localization of AT1R and AT2R determined by immunohistochemistry has shown that AT1 receptors are found in surface epithelium, crypt bases, and lamina propria, whereas AT2R s are mainly in the mesenchymal cells of the lamina propria [244]. The physiologic functions of these receptors are different based on Ang II-mediated activation. AT2R stimulation plays a role in water and sodium absorption from the colon. AT1 receptor-mediated pathway leads to sodium reabsorption, sodium secretion, and colon motility [245, 246].

Components of RAAS have been demonstrated to have a close relationship with the progression and metastasis of CRC. CRC presentation is often associated with advanced tumor stage and liver metastasis [247]. AT1R expression is found to play a pro-tumoral role and has a correlation with liver metastasis and pathological tumor stage [248, 249]. In AT2R knockdown CMT93 cells followed by Ang II dose-dependent treatment, proliferation, invasion, and VEGF secretion were observed which contributed to AT1R stimulation. The same study elucidated that AT2R activation via Ang II treatment in low concentrations is associated with increased VEGF secretion, whereas the high concentration of Ang II resulted in antitumoral effects by inhibition of cell growth and metastasis [249]. Loss of expression of AT2R in advanced and aggressive CRC cells may also be contributed to CRC progression [249, 250]. A study by Namsolleck et al. demonstrated that LP2, the cyclic Ang (1–7) analog which is a specific agonist of AT2 receptor, inhibits the growth of patient-derived xenografts of CRC in mice via Pi3K/AKT/mTOR signaling which results in apoptosis via CDKs. This modified peptide of Ang (1–7) with the N-terminal D-lysin and high specificity, stability and excellent pharmacokinetics, showed antitumor activity against CRC in mice models which also acted synergistically with 5-FU and Erlotinib. [251].

A study on patients with colon adenocarcinoma has shown that Mas Receptor was markedly upregulated in comparison to the non-neoplastic region [250, 253]. However, treatment of human colon adenocarcinoma expressing MasR with Ang 1–7 exhibited no change in cell cycle events and proliferation [253]. The increase of MasR in cancer development in CRC in comparison to normal tissues suggests a potential role for this receptor that needs to be elucidated.

An in vitro study by Oura et al. indicated that Telmisatan can inhibit the proliferation of HCC and induce cell cycle arrest [254].The use of an AT1R inhibitor (Irbesartan) and an ACE inhibitor (Captopril) in a mice model of CRC liver metastasis further elucidated the role of Ang II/AT1R signaling in the induction of metastasis [169]. In fact, the blockade of AT1R has shown to promote anti-tumor effects in KCs in the early stages of tumor growth [169, 255]. Furthermore, the study of RAAS inhibition by an Ang II receptor blocker, Valsaratan, in CRC mouse models demonstrated CRC cell growth suppression and inhibition of cell migration by perturbation of MMP-2 and MMP-9 [256]. Clinical data analysis also represents an association between the use of RAAS inhibitors and better outcomes in CRC [173, 257–259]. A retrospective analysis of clinicopathological data of CRC patients exhibited that ACEI/ARB treatment may reduce tumor recurrence in left-sided CRC and early-stage CRC [260]. However further investigation and prospective clinical trials is required to establish the clinical significance of ACEIs and ARBs in CRC treatment and recurrence.

### RAAS components in gastric cancer (GC)

Localization of AT1R and AT2R in the epithelium and lamina propria is suggested to be contributed to the functional impact of these elements in physiological conditions. AGT and Renin are both expressed by resident mesenchymal cells in lamina propria which contribute to the formation of the principal mediator, Ang II, in the tissue [261] [262].

The association of RAAS components with gastric cancer has been reported previously [263]. The most studied RAAS axis in gastric cancer is the ACE/Ang II/AT1R which is found to elaborate on many mechanisms involved in cancer progression. The expression of Ang II, AT1, and the ACE activity in gastric cancer patients is upregulated in comparison to healthy tissues [264]. Ang II is reported to have a role by its impact on the induction of MMP-2 and MMP-9 expression in human gastric cancer cells which contribute to tumor invasion and metastasis [204]. Ang II also has the potential to increase proliferation and impair apoptosis by ERK1/2 and NF-κB activation and overexpression. Furthermore, AT1 receptor blockade confirms the anti-proliferative and tumor growth inhibition role of Ang II/AT1R-induced signaling in gastric cancer [265]. In another study, Candesartan significantly suppresses tumor proliferation and fibrotic changes in mice subcutaneous xenograft model of gastric cell line with human peritoneal mesothelial cells (HPMC). This is suggested to be by TGF-β1-induced EMT change and stromal fibrosis by Ang II/ATR signaling which is suppressed by blockade of AT1 receptor. [266].

In a study on GC patients, the local expression of AT2R is reported [267]. However, its role in GC has been controversial. A study on human GC cell lines demonstrated that in vitro blockade of the AT2 receptor reduced invasive abilities of the tumor cells with no effect on cell number [268]. In another study on gastric cancer patients, it is demonstrated that the AT2 expression along with AT1 and ACE was increased compared to healthy tissues [264]. In the contrary, in another study, AT2R expression has been demonstrated to be significantly down-regulated in GC patients. Moreover, activation of AT2R decreases migration and invasion of cancerous cells in an intraperitoneal carcinomatosis xenograft mouse model. It also decreases tumor formation and increases mouse survival in an in vivo model [269]. Thus, further investigation on the role of AT2R in GC requires to be determined considering the counter-regulatory effect on the AT1 receptor which is implied in other cancer cells.

### RAAS components in pancreatic cancer

 [270]. The expression of RAAS components has been found in different cell types of the pancreas including pancreatic ducts, endothelial, acinar, and stellate cells [270–272]. It has been demonstrated that RAAS components have roles in the regulation of many physiological mechanisms and are responsive to pathophysiological conditions including pancreatic cancer [273–275].

It is suggested that RAAS components play an important role in pancreatic cancer growth and progression [276, 277]. Over-expression of AT1R mRNA and protein has been observed in pancreatic cancer tissue compared to normal tissues. This study also demonstrated that AT1R selective antagonist (L-158,809) suppresses the growth of human pancreatic cancer which suggests the role of Ang II and AT1R in pancreatic cancer [277]. In a study on miR-410 role in pancreatic cancer, it was demonstrated that AT1R mRNA is the direct target of this microRNA which also suppresses AT1R expression [278]. Furthermore, it was suggested that miR-410 suppresses angiogenesis partly through inhibiting the ERK1/2 pathway via AT1R suppression. It is also demonstrated that AT1R knockdown results in suppression of cell proliferation and invasion in pancreatic cancer cells [278].

Inflammation is one of the key steps in the development of pancreatic cancer. Studies have found that fibrosis is involved in the transformation from pancreatitis to pancreatic cancer and further tumor growth and metastasis [279, 280]. Pancreatic stellate cells are the key mediators of fibrosis [281]The role of Ang II in pancreatic inflammation and fibrosis has been investigated in WBN/Kob rats. It was demonstrated that Ang II stimulates up-regulation of α-SMA and TGF-β which are the known fibrosis markers that are expressed by activated pancreatic stellate cells [276, 282]. ROS accumulation is enhanced following chronic inflammation and pancreatic fibrosis compared to normal tissues. Given that Ang II mediates ROS generation [283], oxidative stress induced by Ang II signaling promotes pancreatic fibrosis [276].

Pancreatic ductal adenocarcinoma (PDAC) is the most common and also most lethal pancreatic cancer type [284] The study on human PDAC specimens indicated that AT1R expression is significantly higher than in normal pancreas, whereas AT2R expression level in neoplastic ductal epithelium was slightly lower than in normal pancreas [285]. This study also demonstrated that selective AT2R agonist treatment using murine PDAC grafts attenuates the growth of tumor in volume and weight by inducing apoptosis in PDAC cells [285]. This suggests the potential benefit of AT2R agonists in combination with AT1R antagonists in PDAC treatment.

The expression of the ACE2/Ang 1–7/MasR axis is confirmed in the pancreas of an animal model of Severe Acute Pancreatitis (SAP) [286]. The study on in vitro model of pancreatitis demonstrated that the ACE2/Ang 1–7/MasR axis significantly suppresses the p38 MAPK/NF-κB signaling pathway in mouse acinar cells which inhibits pancreatitis [287]. Localization and expression of ACE2 in human pancreatic cells particularly in beta cells, have also been reported [288]. Although the protective and anti-inflammatory effects of the ACE2/Ang 1–7/MasR axis have been recognized in rodents and in vitro studies of the pancreas [287, 289–291], further investigation of this axis is needed to shed light on its exact role in human pancreatic cancer.

Common ACE inhibitors and ARBs have emerged as promising therapeutic strategies in PDAC. In a study by Zhou et al. using a high-throughput screening platform of the Gemcitabine-resistant PDOs (patient-derived organoids), reported that Ibesartan, reduces resistance to chemotherapy. In vitro and in vivo studies demonstrated that ibesartan sensitizes PDAC tumors to chemotherapy. The mechanism underlying gemcitabine-sensitivity is found to be by suppressing c-Jun expression via repressing the activation of Hippo/YAP1/TAZ signaling pathway. It is revealed that tumoral c-Jun significantly enhances tumoral iron metabolism and promotes cancer cell stemness in PDAC which can be inhibited by ibesartan [292].

Several clinical trials have investigated the role of losartan in combination with chemotherapy for PDAC. A phase II trial found that adding losartan to gemcitabine and nab-paclitaxel improved progression-free survival and overall survival compared to chemotherapy alone in patients with locally advanced PDAC. Another retrospective study of PDAC patients undergoing resection indicated that RAAS inhibitor users compare to non-users have a prolonged overall survival [293]. Losartan was found to be an Extra cellular matrix (ECM) modulator by inhibition of TGFβ1 expression, collagen I and hylouronan synthesis in cancer associated fibroblasts [276], while losartan and other RAAS inhibitors have shown promises in preclinical and clinical studies, more research is needed to fully understand their potential as a treatment option [294].

### RAAS components in Hepatocellular Carcinoma (HCC)

Liver cancer comprises different types of hepatic neoplasms with hepatocellular carcinoma (HCC) as the most common type of liver cancer, representing more than 80% of the cases [295]. The pivotal role of RAAS in HCC development and other liver diseases such as liver fibrosis has been documented [296, 297]. Based on findings, local RAAS components are all present in the liver tissues and in its different cell types including hepatocytes, cholangiocytes, and hepatic Kupffer cells [252, 298, 299]. Local RAAS function in the liver is crucial under physiological and pathophysiological conditions as it plays a role in cellular functions including metabolic processes, regulation of cell proliferation, angiogenesis, and apoptosis [300–302].

Many studies have shown that Ang II/AT1R activation promotes cell proliferation, angiogenesis, inflammation, fibrosis, and ECM remodeling in HCC which is associated with cancer progression [303–305]. A study on HepG2 cells demonstrated that Ang II induces cell proliferation by AT1R/Raf/ERK1/2 signaling pathway. In support, cell treatment with Candesartan (AT1R blocker), Sorafenib (Raf inhibitor) and ERK inhibitor PD98059 indicated significant inhibition of cell proliferation [306]. Another study on HCC cell lines evaluates the downstream effects of Ang II/AT1R signaling in HCC. Their data proposed that the AT1/PKC/NF-κB pathway enhances the proliferation and inflammation induced by Ang II in HCC cells. Based on the results, stimulation of AT1R activates Protein Kinase C (PKC) signaling which plays an important role in cell proliferation and migration of HCC cells [307]. It is further demonstrated that the PKC plays a key role in NF-κB signaling pathway [303]. NF-κB signaling maintains the inflammatory response of HCC cells and also contributes to cancer progression and survival as well as enhancing cell proliferation [303, 308]. The role of RAAS in HCC was highlighted by targeting the AT1R and by mean of microRNA-152 (miR-152). The results suggested that miRNA-152 expression resulted in down-regulation of AT1R, expression of vimentin, N-cadherin and the number of invaded and migrating cells [309].

Ang II/AT1R activation also induces angiogenesis by activating signaling pathways including AT1/JAK2/STAT3/SOCS3 [310] which confirms the results from a study on subcutaneous xenograft tumors derived from HCC cells in nude mice treated with Candesartan. In this experiment, Ang II promoted VEGF through AT1R activation. Moreover, microvessel density (MVD) as an angiogenic parameter of tumors is positively correlated with AT1R and VEGF expression. Candesartan is able to significantly reverse these effects and exhibit anti-angiogenic effects [94]. An In vivo study experimenting the role of Ang-II/AT1R signaling in metastasis revealed that Ang II upregulates VCAM-1 via the p38/MAPK pathway in HCC cells which is related to adhesion to endothelial cells and promotes metastasis [311].

Most HCCs are developed in the context of liver fibrosis, with 90% of the cases developing on the background of liver cirrhosis [312, 313]. In this regard, Hepatic Stellate Cells (HSCs) play a key role [314]. The activation of HSCs along with extracellular deposition are the key steps in liver fibrosis [315]. RAAS is one of the main mediators of HSCs activation [316, 317]. Ang II is known to be the central effector in activating the HSCs to produce pro-fibrotic markers such as TGF-β1 and promote inflammation and oxidative stress [316, 318]. In vivo investigation of RAAS inhibition by an ARB (Candesartan) and an ACEI (Perindopril) on pig serum-induced liver fibrosis model significantly attenuated fibrosis development [319]. In many studies, RAAS blockade by AT1R inhibitors or ACEIs led to reduced liver fibrosis by inhibition of HCSs through suppression of AT1R/TGF-β/Smad and AT1R/TGF-β/MAPK signaling pathways which further decrease the generation of ECM [320–322]. Supporting data suggested that using losartan, enhances anti-PD-1-triggered HCC regression in a mouse model of HCC [323].

The role of AT2R in various types of cancer has been controversial. AT2R expression in HCC cell lines is found to inhibit proliferation via down-regulation of CDK4 and cyclinD1. It is also demonstrated that AT2R induces apoptosis via MAPK pathway, Caspase-3 and Caspase-8 signaling. On the contrary, an in vivo study indicated that while high levels of AT2 receptor induce apoptosis, moderate expression of this receptor promotes cell proliferation and increases tumor growth. This implicates that the level of AT2R expression mediates different effects on HCC cells [324].

ACE2, in another arm of the RAAS was found to have prognostic value in HCC. The immunohistochemical analysis of ACE2 expression in an HCC microarray revealed that ACE2 expression was down-regulated in HCC tissues compared to normal liver. Secondly, in vitro and in vivo experiments showed that ACE2 overexpression inhibits HCC tumor growth through blocking the aerobic glycosis in HCC via Ang (1–7)/Mas Receptor axis with the downstream signaling cascade of p-SHP2/ROS/HIF1α axis [325]. The beneficial effects of the Ang 1–7/MasR axis have been previously reported in different types of cancer cells [326, 327]. Ang 1–7 inhibits proliferation and induces apoptosis by caspase-3 activity in vitro and in vivo. Another antitumor impact of Ang 1–7/MasR in HCC is the antiangiogenic effect which is mediated by down-regulation of VEGF-A secretion. Ang 1–7 is also proposed to play an important role in the expression of RAAS receptors. Ang 1–7 has been shown to down-regulate AT1R and upregulate AT2R along with MasR expression in vivo and in cultured H22 cells. It is suggested that Ang 1–7 may exert its antitumor effects by both MasR and AT2R activation and antagonizing AT1R effects [328].

### RAAS components in esophagus cancer

Previous studies confirmed the presence of local RAAS in human esophageal mucosa. Ang II receptors and ACE are expressed in the human esophageal epithelium. The physiological actions of RAAS in esophageal mucosa are suggested to be by regulation of the ion transport [329] and also the esophagus contraction [330]. The role of RAAS the in pathophysiology of many esophagus malignancies such as esophageal adenocarcinoma and Esophageal Squamous Cell Carcinoma (ESCC) has been demonstrated [331, 332].

It is demonstrated that AT1R overexpression is significantly associated with higher T classification and overall worse prognosis. In vitro and In vivo studies indicated that ESCC cell proliferation is stimulated by Ang II in a dose-dependent manner. The mechanism underlying this effect is suggested to be via Ang II/AT1R signaling and further activates the mTOR pathway [332, 333]. In support, findings suggested that Telmisartan inhibited human esophageal adenocarcinoma (EAC) cell proliferation and tumor growth by inducing cell-cycle arrest via the AMPKα/mTOR pathway in EAC cells [332]. In vivo and In vitro studies also demonstrated the role of ACE inhibitors and ARBs in the suppression of angiogenesis by inhibition of VEGF expression [334, 335]. However, RAAS inhibition by ARBs and ACEIs as a therapeutic strategy in oesophgeal cancer has not shown prognostic effects. The retrospective study of patients with EAC and ESCC found no difference in overall survival and disease-free survival between users of RAAS inhibitor and non-users [336, 337].

The study of AT2R expression in ESCC cells indicated that overexpression of AT2R is associated with high T classification and AT1R expression. Moreover, the use of an AT2R antagonist, PD123319, exhibited no effect on ESCC cell proliferation [333]. Hence, the role of AT2R in esophageal cancer is still not well recognized.

The Ang 1–7/MasR axis is also investigated in Esophageal cancer. Findings suggested the antiproliferative effects of Ang 1–7/MasR signaling in vitro [338]. The study of the clinical impact of MasR on ESCC patients receiving curative esophagectomy demonstrated low MasR expression being correlated with a higher recurrence rate, poor disease-free, and overall survival. [338]. It is suggested that Ang 1–7/MasR axis could be both recognized as a diagnostic tool and an adjuvant therapy.

## Conclusion

RAAS as a fundamental system is proven to have a potential role in proliferation signaling, cell migration, angiogenesis, invasion, and metastasis. Expression of RAAS receptors has been identified in various tumors, proposing these new-in receptors in cancer as novel markers for the early detection of malignancies. Additionally, Ang II, as one of the major peptides of RAAS, is demonstrated to prompt cancer metastasis by affecting and upregulating metastatic hallmarks. Thus, the use of drugs that affect RAAS may be proposed as an adjuvant treatment option in cancer. Prophylactic use of these agents may also aid in the prevention of tumor formation.

Considering the somewhat safe profile of RAAS inhibitors propose the use of RAAS modulators as a promising strategy in preventing and curing cancer as well as metastasis.

## Data Availability

Not applicable.
